# Anti-apoptotic effect of the Shh signaling pathway in cigarette smoke extract induced MLE 12 apoptosis

**DOI:** 10.18332/tid/109753

**Published:** 2019-06-06

**Authors:** Jinhua Li, Dandan Zong, Yan Chen, Ping Chen

**Affiliations:** 1 Department of Respiratory Medicine, The Second Xiangya Hospital, Central South University, Changsha, China; 2 Research Unit of Respiratory Disease, Central South University, Changsha, China; 3 Diagnosis and Treatment Center of Respiratory Disease, Central South University, Changsha, China

**Keywords:** cigarette smoke extract, apoptosis, alveolar type II epithelial cells, Shh signaling pathway

## Abstract

**INTRODUCTION:**

Many studies have shown that COPD is associated with apoptosis of bronchial or alveolar epithelial cells. Alveolar type II epithelial cells (AECII) play an important role in the pathogenetic process. Cigarette smoke extract (CSE) can induce apoptosis of AECII. The Sonic hedgehog (Shh) pathway is involved in many adult lung diseases. We aimed to verify the anti-apoptotic effect of Shh in the AECII apoptosis induced by CSE.

**METHODS:**

Mouse lung epithelial type II cells, MLE 12, were treated by 5% CSE for 24 hours. Apoptosis was measured using flow cytometry and expression of the anti-apoptotic factor BCL-2. The role of the hedgehog pathway in cell apoptosis was assessed by real-time RT-PCT and western blotting to measure the expression of Sonic hedgehog, Patched 1, and Gli1. Recombinant mouse Sonic hedgehog was used to overexpress the Shh pathway.

**RESULTS:**

CSE could induce MLE 12 apoptosis. Sonic hedgehog, Patched 1 and the Gli1 were decreased in the CSE induced MLE 12 apoptosis. Overexpression Shh could partially reverse the CSE induced apoptosis.

**CONCLUSIONS:**

Activation of the Shh pathway may relieve the CSE induced MLE 12 apoptosis.

## INTRODUCTION

Chronic obstructive pulmonary disease (COPD) has become an increasingly serious threat to human health because of its high prevalence and related disability and mortality^1^. The overall prevalence of spirometry-defined COPD was 8.6%, accounting for 99.9 million people with COPD in China^2^. But the pathogenesis remains poorly understood. Smoking exposure is the risk factor most related to COPD. Many studies have shown that COPD is associated with apoptosis of bronchial or alveolar epithelial cells^3-5^. Alveolar type II epithelial cells (AECII) play an important role in the pathogenetic process. Cigarette smoke extract (CSE) can induce apoptosis of AECII^6^. Also, the endothelial cells^7^ and smooth muscle cells^8^ can be induced by apoptosis by CSE. The apoptosis of epithelial cells is closest to the pathogenesis of COPD *in vivo* and most widely studied.

Increasing evidence suggests that the Sonic hedgehog (Shh) pathway is involved in many adult lung diseases such as pulmonary fibrosis, COPD, asthma, and lung cancer^9^. The hedgehog (Hh) family includes Shh, Indian hedgehog (Ihh) and Desert hedgehog (Dhh)^10^. Shh is the most broadly expressed HH ligand. The Shh signaling pathway involves two transmembrane proteins on receiving cell, Patched (Ptc), and Smoothened (Smo), which is the signaling component of the SHH-receptor complex^10^. In the nucleus of a responding cell, zinc-finger transcription factors of the Gli family (GLI1–3) act at the last step of the SHH-signal-transduction pathway^10^. Many studies show the anti-apoptotic effect of the Shh signaling pathway^11-14^. Moreover, a recent study has shown that the apoptosis of AECII induced by hyperoxia-induced oxidative stress-related injury was via the inhibition of the Sonic hedgehog pathway^15^.

Few studies have investigated the anti-apoptotic effect of Shh in the CSE induced AECII apoptosis. In this study, we tested the hypothesis that Shh was inhibited in the CSE induced AECII apoptosis.

## METHODS

### Cell culture

Mouse lung epithelial type II cells, MLE 12, were purchased from ATCC. These cell lines were cultured in the recommended medium supplemented with 5% fetal bovine serum and maintained at 37°C in a humidified atmosphere with 5% CO_2_. The medium was replaced every 2 days.

### Preparation of CSE

Half a cigarette (Marlboro, China) was smoked through a 0.22 mm filter to remove particles and bacteria into a vessel containing 20 mL of 5% fetal bovine serum and was considered as the starting solution of CSE. The pH of the resulting CSE solution was 7.4. CSE was prepared fresh and before each experiment and diluted to 1%, 2.5%, 5% and 7.5% as working concentrations.

### Apoptosis by flow cytometry

MLE 12 cultured in a six-well plate were treated with CSE (0%, 1%, 2.5%, 5%, and 7.5%), CSE+ recombinant Shh (150 ng/mL, Recombinant Mouse Sonic Hedgehog/Shh (C25II) N-Terminus, R&D Systems, USA) and cyclopamine (15 umol/L, APExBIO, USA) for 24 h. One well of cells (about 1–5×10^5^ cells) were then harvested, washed and resuspended in phosphate-buffered saline (PBS). Apoptotic cells were identified using an annexin V-fluorescein isothiocyanate (FITC)/propidium iodide (PI) cell apoptosis kit (KeyGEN BioTECH, China) according to the manufacturer’ s protocol. Briefly, the cells were washed and subsequently incubated with 500 μL of 1×binding buffer containing 5 μL of annexin V-FITC and 5 μL of PI for 15 min in the dark. Apoptosis was then analyzed by flow cytometry (BD Biosciences). The early apoptosis determines the percentage of apoptosis. Each experiment was repeated three times.

### Real-time RT-PCR

MLE 12 were treated with CSE (0%, 5%) for 24 h. RNA was collected through TRIzon reagent (Cwbio, China) according to the instructions. Reverse transcription of the first strand cDNA was operated using RevertAid First Strand cDNA Synthesis Kit (Thermo Fisher Scientific, USA). Real-time quantitative PCR was performed using All-in-One^TM^ Qpcr Mix (GeneCopoeia^TM^) on a CFX96™ PCR machine (Bio-Rad, Hercules, CA, USA). All procedures were conducted according to the manufacturer’s instructions. Beta actin was used as the housekeeping gene. The comparative C(T) method was used to analyze real-time PCR data^16^. Each experiment was performed twice in triplicate.

### Western blotting

MLE 12 were treated with CSE (0%, 2.5%, 5%, and 7.5%) for 24 h. Cells were harvested in RIPA cell lysis buffer supplemented with protease inhibitors (Merck, Germany), and protein concentrations were determined using the BCA protein assay. Protein extracts (20 μg) were separated by SDS-PAGE using 12% and 8% polyacrylamide gels and then transferred to polyvinylidene difluoride (PVDF) membranes. Membranes were blocked with 1*TBST containing 5% skim milk, incubated overnight at 4°C with primary antibodies against Shh (proteintech, USA), Gli1 (Abcam, UK), Ptch1 (proteintech, USA), BCL-2 (CST, USA) and β-actin (proteintech, USA) then incubated with a horseradish peroxidase-conjugated goat anti-rabbit IgG antibody (Proteintech, USA) for 1.5 h at room temperature.

Immunoreactivity was detected using an enhanced chemiluminescence kit according to the manufacturer’s instructions. Protein expression levels were normalized against β-actin expression.

### Statistical analysis

Results are expressed as mean ± standard deviation. Variances among at least three groups were assessed using one-way analysis of variance. A p-value of 0.05 was considered statistically significant. Data were analyzed using SPSS version 18.0 for Windows (SPSS Inc., Chicago, IL, USA).

## RESULTS

### Apoptosis after CSE treatment

**Figure 1:**
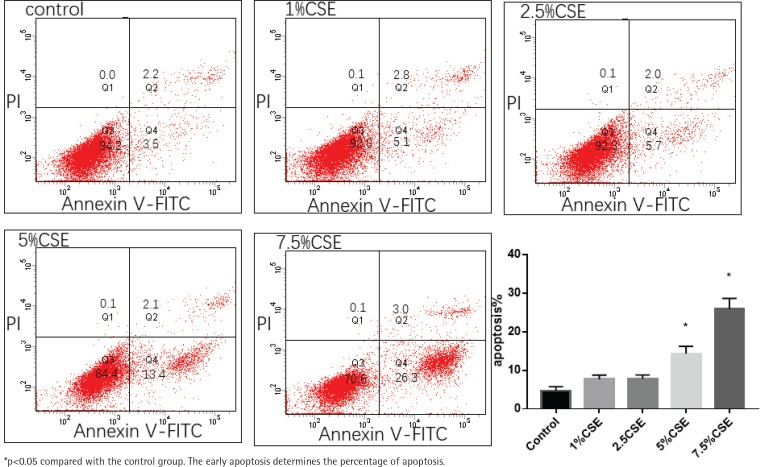
Effect of varying concentrations of CSE induced apoptosis in MLE 12

MLE 12 were treated with varying concentrations of CSE (0%, 1%, 2.5%, 5%, and 7.5%) for 24 h, before evaluation of apoptosis by flow cytometry. After CSE treatment, there was significantly increased apoptosis in the 5%CSE group (14.3±1.9%) and 7.5%CSE group (25.83±2.78%), compared to the control group (4.7±1.05%), while there was no significant increased apoptosis in the 1%CSE group (7.63±1.1%) and 2.5%CSE group (7.73±1.04%) compared to the control group. It showed that as the concentration of CSE increases, the apoptosis of MLE 12 increases (Figure 1).

### Effect of CSE on mRNA expression of the Shh pathway

**Figure 2:**
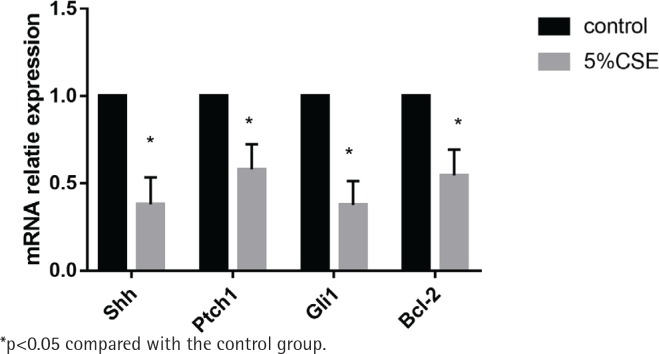
Effect of CSE on mRNA expression of Shh pathway

MLE 12 were treated with and without 5%CSE for 24 h before the detection of mRNA expression of the Shh pathway. It showed that after CSE treatment, the mRNA of the Shh pathway (Shh, Ptch1, Gli1) and the anti-apoptotic factor BCL-2 were decreasing. The results indicate that CSE could inhibit the Shh pathway in MLE 12 and promote apoptosis (Figure 2).

### Effect of CSE on protein expression of Shh pathway

**Figure 3:**
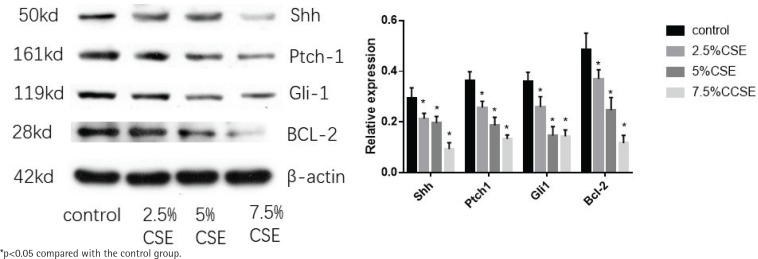
Effect of CSE on protein expression of Shh pathway

MLE 12 were treated with varying concentrations of CSE (0%, 2.5%, 5%, and 7.5%) for 24 h, before western blotting. After CSE treatment, the protein expression of the Shh pathway (Shh, Ptch1, Gli1) decreased and so was the anti-apoptotic factor BCL-2. There was a trend, as the CSE concentration increased, the more the protein of Shh pathway and BCL-2 decreased. The results also indicate that CSE could inhibit the Shh pathway in MLE 12 and promote apoptosis (Figure 3).

### Effect of overexpression of Shh on CSE induced apoptosis of MLE 12

**Figure 4:**
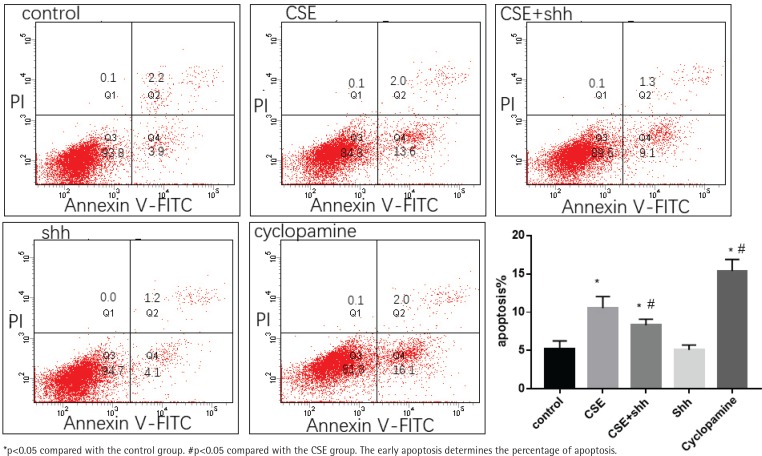
Effect of overexpression of Shh on CSE induced apoptosis of MLE 12

MLE 12 were treated with 5%CSE, CSE+Shh (a recombinant mouse Shh protein), Shh, and cyclopamine (an Shh pathway inhibitor) for 24 h, before evaluation of apoptosis by flow cytometry. The result showed that after treatment with cyclopamine, the apoptosis increased compared with the control group. When treated with CSE+Shh, Shh could partially reverse the apoptosis caused by CSE (Figure 4).

## DISCUSSION

It is known that cigarette smoke exposure is the major risk factor of COPD. Apoptosis is an important mechanism in COPD^17^. The most important pathogenesis of COPD is abnormal apoptosis in airway epithelium, due to long-term exposure to cigarette smoke^18^. Numerous studies have shown the epithelial cell apoptosis of COPD *in vivo* and *in vitro*. MLE 12 were used as the AECII *in vitro* in many studies^19^. In our study, we found that CSE could induce apoptosis of MLE 12 that is positively correlated with the CSE concentration.

However, the mechanism to apoptosis is not clear. We investigated the anti-apoptotic effect of the Shh signaling pathway in the CSE induced apoptosis of MLE 12. In this study, the mRNA of the Shh pathway decreased after CSE treatment and the protein of Shh signaling pathway also deceased, which is negatively correlated with the CSE concentration. In many other studies, Shh pathway shows its anti-apoptotic effect. In a study of breast cancer^20^, it was found that inhibition of the Shh pathway induces cell apoptosis. In a renal ischemia/reperfusion study^13^, the activating Shh pathway leads to a decrease in apoptosis. In another study, it was demonstrated that components of Shh signaling, Patched and Gli3, are expressed in human platelets, consistent with the existence of functional Hedgehog signaling in these cells^14^. The studies of the anti-apoptotic effect of Shh pathway in pulmonary diseases are relatively few. In a study of lung cancer stem cells^21^, inhibition of the Shh pathway was found to cause increased apoptosis. In idiopathic pulmonary fibrosis (IPF), the Shh pathway is activated in IPF lungs and the Shh pathway may play a role in increasing the proliferation^22^. There are no studies that investigated the anti-apoptotic effect of Shh pathway in COPD or emphysema. Our data show that the Shh pathway may play a role in the anti-apoptotic effect of CSE induced apoptosis in MLE 12. Also, our data show that after treatment with recombinant mouse Shh protein, the apoptosis can be partially reversed and cyclopamine, an Shh pathway inhibitor, can increase MLE 12 apoptosis. Shh protein may promote the proliferation of the cells to compete for the apoptosis. In a previous study, the Shh pathway was mostly studied in embryonic lung development^9^ with the mechanism possibly promoting the proliferation of the stem cells. Overexpression of Shh protein can help repair lung injury by increasing the lung stem cells^23^. Our data show similar results as this previous report. Based on existing evidence and our current data, we propose that the Shh pathway plays a vital role in CSE induced MLE 12 apoptosis.

In this cell experiment, we investigated whether over-expression of the Shh pathway inhibits the AECII apoptosis. We found that Shh may be able to reverse the progress of COPD. In a future study, we intend to verify the hypothesis in COPD animal models and COPD patients. The ultimate goal is to find a new drug to treat COPD.

## CONCLUSIONS

To our knowledge, this is the first study to indicate that activation of the Shh pathway may relieve CSE induced AECII apoptosis, which is a promising therapeutic target. Further studies and a greater understanding of the changes that occur in the alveoli are required to elucidate how Shh inhibition and AECII apoptosis are involved in the pathology of COPD.

## CONFLICTS OF INTEREST

The authors have completed and submitted an ICMJE form for disclosure of potential conflicts of interest and they declare that they have no competing interests, financial or otherwise, related to the current work. All the authors report grants from National Natural Science Foundation of China and grants from Natural Science Foundation of Hunan Province, China, during the conduct of the study.
